# New records of three vent-endemic scale worms (Annelida, Polynoidae) from the northern Central Indian Ridge fill regional distributional gaps

**DOI:** 10.3897/zookeys.1287.198002

**Published:** 2026-08-05

**Authors:** Won-Kyung Lee, Se-Joo Kim

**Affiliations:** 1 Metabolic Regulation Research Center, Korea Research Institute of Bioscience and Biotechnology, Daejeon, 34141, Republic of Korea Metabolic Regulation Research Center, Korea Research Institute of Bioscience and Biotechnology Daejeon Republic of Korea; 2 KRIBB School, University of Science and Technology, Daejeon, 34113, Republic of Korea KRIBB School, University of Science and Technology Daejeon Republic of Korea

**Keywords:** *COI*, contract areas, DNA barcoding, Lepidonotopodini, morphology, nCIR

## Abstract

The tribe Lepidonotopodini (Polychaeta, Polynoidae) consists of 61 species in 12 genera. Species in this tribe lack eyes and lateral antennae and exhibit pronounced sexual dimorphism; some have external branchiae. Here, we report new records of three species of Lepidonotopodini—*Cladopolynoe jiaolongae*, *Photinopolynoe kaireiensis*, and *Themis longqiensis*—from hydrothermal vents on the northern Central Indian Ridge (nCIR) based on morphological characteristics and *COI* barcodes. Morphological characters missing in the original description papers are also provided here to complement and refine these species’ diagnoses. The *COI* barcodes of all three species showed low intraspecific divergence (0.00–1.55%), substantially lower than interspecific divergence within the genus (7.77–24.47%). These findings expand the known distribution ranges and clarify the species boundaries of these taxa in nCIR vent fields. Consequently, this study contributes to a better understanding of regional biodiversity and helps establish biological baselines for environmental management within the contract areas.

## Introduction

Polynoidae Kinberg, 1856, is a species-rich family of marine benthic polychaetes that plays a crucial ecological role (Rouse and Pleijel 2001; Díaz-Castañeda and Reish 2009; Hourdez 2022; Rouse et al. 2022). Within this family, the tribe Lepidonotopodini Pettibone, 1983 includes 61 species in 12 genera that exclusively inhabit deep-sea chemosynthetic-based ecosystems globally (Read and Fauchald 2026). Lepidonotopodini exhibits notably high species diversity at the tribe level compared to other vent-associated taxa (Leignel et al. 2017; Hatch et al. 2020; Chan et al. 2021).

To date, seven species have been reported from the Indian Ocean. These are distributed across different ridge systems: *Branchipolynoe longqiensis* Zhou et al., 2017 and *Stratigos bipapillata* (Zhou et al., 2018) on the Southwest Indian Ridge (SWIR) and Central Indian Ridge (CIR); *B. onnuriensis* Kim et al., 2022 on the CIR; *Cladopolynoe jiaolongae* (Han et al., 2023) on the SWIR and Carlsberg Ridge (CR); *Photinopolynoe kaireiensis* (Han et al., 2023) on the CIR and CR (Zhou et al. 2018, 2022; Han et al. 2023; Lee and Kim 2024); *Themis longqiensis* (Han et al., 2023) on the SWIR; and *T. pettiboneae* (Han et al., 2023) on the SWIR, CIR, and CR.

The geographic distribution of these deep-sea species remains poorly understood due to sampling difficulties in remote deep-sea environments with limited accessibility. Also, most polynoids easily lose fragile diagnostic structures (e.g. elytra, dorsal cirri, body segments), resulting in incomplete specimens (Han et al. 2023). In the northern CIR (nCIR), despite the presence of multiple hydrothermal vent fields, biodiversity data remain scarce besides the well-studied Onnuri vent field (OVF). Therefore, continued sampling and redescription of Lepidonotopodini polynoids are crucial to refine species diagnoses, clarify taxonomic relationships, and resolve regional distribution patterns.

Here, we report new records of three Lepidonotopodini polynoids from the nCIR, including six vent fields (Cheoeum, Maru, Onbada, Onnare, Onnuri, and Saero). Their morphological diagnoses were revised based on comparison with type specimens, and genetic divergence was analyzed using cytochrome c oxidase subunit I (*COI*) sequences. These findings significantly expand the known distribution ranges of these taxa in vent fields of the nCIR. Accordingly, this study contributes to a better understanding of regional biodiversity and provides valuable biological baseline data for environmental management in deep-sea mining contract areas.

## Materials and methods

### Sample collection

**Figure 1.**
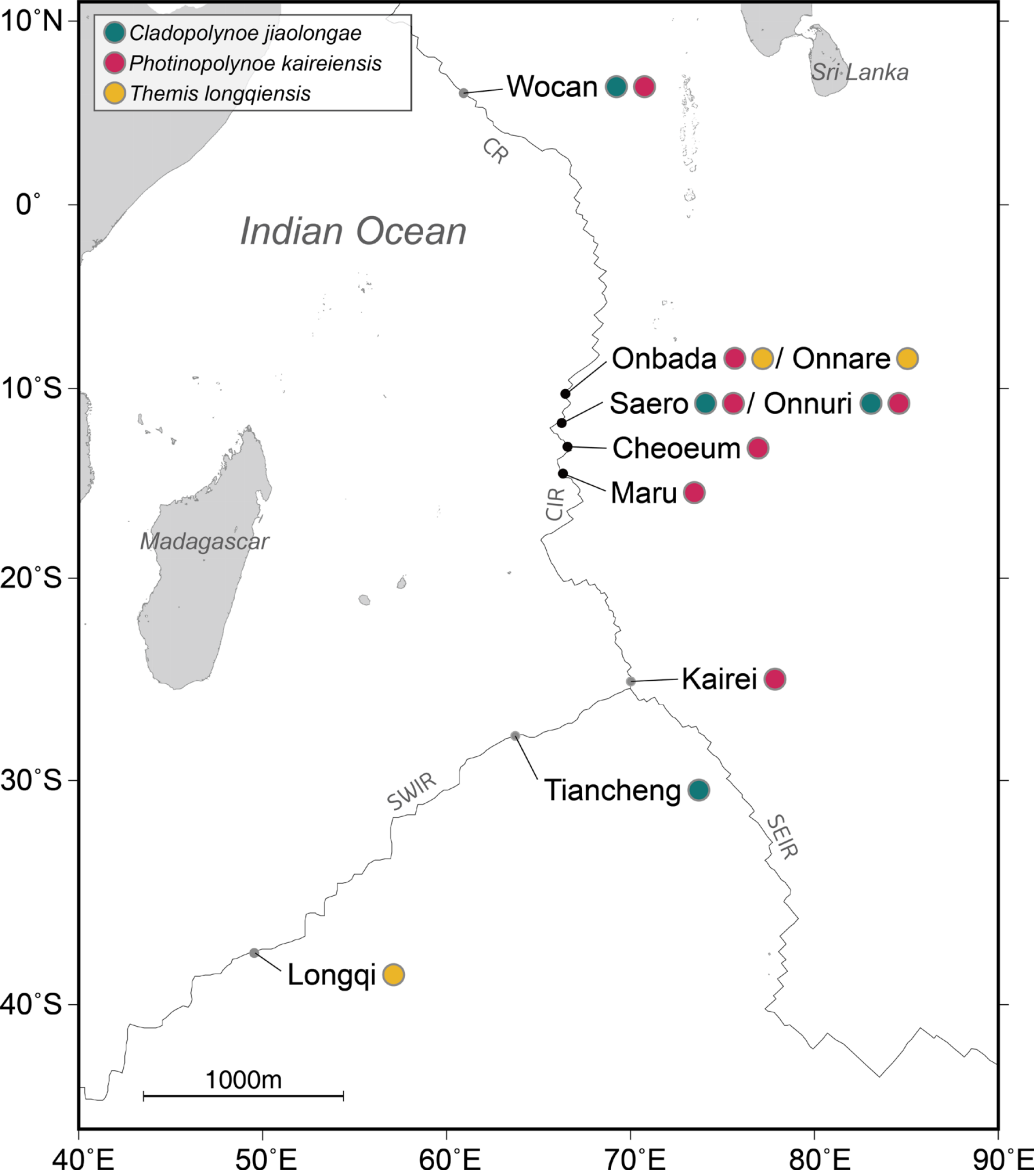
Map displaying the geographic distribution of *Cladopolynoe jiaolongae*, *Photinopolynoe kaireiensis*, and *Themis longqiensis* in the Indian Ocean. Black dots indicate sampling locations from this study and gray dots indicate records from previous studies. Some closely located vent fields (<10 km apart) are marked as a single point. CIR, Central Indian Ridge; CR, Carlsberg Ridge; SWIR, Southwest Indian Ridge; SEIR, Southeast Indian Ridge.

Specimens of *C. jiaolongae*, *P. kaireiensis*, and *T. longqiensis* were collected from hydrothermal vents in the nCIR during cruises in 2023 and 2024 on R/V Isabu, using a suction sampler and scoop mounted on the ROV *ROPOS* (Fig. 1, Table 1). Upon collection, a piece of the elytron or parapodium from each specimen was dissected and preserved in 99% ethanol for molecular analysis.

**Table 1.** Sampling information of newly obtained Lepidonotopodini species from the northern Central Indian Ridge and their GenBank accession numbers of *COI* barcodes sequenced in this study.

**Species**	**Specimen Voucher**	**Vent field**	**Depth (m)**	**Date**	**Latitude, Longitude**	**GenBank Acc. No**.
*Cladopolynoe jiaolongae*	KRIBB314001–KRIBB314002	Onnuri	2,009	Apr. 1, 2023	11°24.9'S, 66°25.4'E	PZ334608–PZ334609
	KRIBB314003– KRIBB314010	Saero	3,273	May 6, 2024	11°19.6'S, 66°26.9'E	PZ334610–PZ334617
*Photinopolynoe kaireiensis*	KRIBB315001– KRIBB315002	Maru	2,256	May 1, 2024	15°31.9'S, 67°17.9'E	PZ334618–PZ334619
	KRIBB315003	Cheoeum	3,038	May 3, 2024	12°37.1'S, 66°7.6'E	PZ334620
	KRIBB315004– KRIBB315009	Onnuri	2,013	May 5, 2024	11°24.9'S, 66°25.4'E	PZ334621–PZ334626
	KRIBB315010– KRIBB315012	Saero	3,267	May 6, 2024	11°19.7'S, 66°26.9'E	PZ334627–PZ334629
	KRIBB315013– KRIBB315018	Onbada	2,532	May 7, 2024	9°48.9'S, 66°40.6'E	PZ334630–PZ334635
*Themis longqiensis*	KRIBB316001	Onnare	2,991	Apr. 3, 2023	9°47.4'S, 66°41.9'E	PZ334636
	KRIBB316002– KRIBB316003	Onbada	2,563	Apr. 4, 2023	9°48.9'S, 66°40.6'E	PZ334637–PZ334638

The entire bodies of all specimens were preserved in either 10% neutral buffered formalin (NBF) or 70% ethanol for morphological studies. The specimens fixed in 10% NBF were rinsed with distilled water after a day and then transferred to 70% ethanol. All newly obtained specimens were deposited at Korea Research Institute of Bioscience and Biotechnology (KRIBB), Daejeon, Korea, each with unique specimen vouchers.

### Morphological examination

For morphological observations, all specimens were examined under a stereomicroscope (Stemi 508; Carl Zeiss, Jena, Germany) and some were examined under an optical microscope (Olympus BX43F; Olympus, Tokyo, Japan). Photographs of key morphological features were taken using a digital single-lens reflex (DSLR) camera (EOS 5D Mark IV; Canon, Tokyo, Japan) and a colour camera (Axiocam 208; Carl Zeiss, Oberkochen, Germany) mounted on a stereomicroscope. Images were processed using ZEN 3.3 blue edition (Carl Zeiss, Jena, Germany) and Helicon Focus v. 7 (Helicon Soft Ltd, http://www.heliconsoft.com), and edited using Adobe Photoshop 2022 (Adobe, San Jose, CA, USA). The identification of *C. jiaolongae*, *P. kaireiensis*, and *T. longqiensis* specimens was based on Han et al. (2023).

### DNA barcoding

A small piece of elytron or parapodium preserved in ethanol was used to extract total genomic DNA using an AccuPrep® Genomic DNA Extraction Kit (Bioneer, Daejeon, South Korea), following the manufacturer’s instructions. Partial *COI* sequences were obtained using the primers LCO1490 and HCO2198 (Folmer et al. 1994). For each specimen, polymerase chain reaction (PCR) reactions were prepared in a final volume of 20 µL containing 1 µL genomic DNA, 2 µL 10× Ex Taq Buffer (Mg^2+^ plus), 2 µL dNTP mixture (2.5 mM each), 1 µL each primer (10 pmol), 0.2 µL Takara Ex Taq (5 U/µL; Takara Bio, Tokyo, Japan), and distilled water to reach 20 µL. PCR was conducted under the following conditions: initial denaturation at 94 °C for 2 min, followed by 35 cycles of denaturation at 95 °C for 10 s, annealing at 48 °C for 30 s, and extension at 72 °C for 1 min, with a final 5-min extension at 72 °C. PCR products were sent to Macrogen (Seoul, Korea) for Sanger sequencing.

The *COI* sequences of each species retrieved from GenBank were aligned with the new sequences using Geneious Prime v. 2022.2.1 (Biomatters, Auckland, New Zealand). Intraspecific, interpopulation, and interspecific divergence values were calculated using the *p*-distance (Nei and Kumar 2000) method in MEGA X (Kumar et al. 2018).

## Results

**Family Polynoidae Kinberg, 1856**

**Subfamily Macellicephalinae Hartmann-Schröder, 1971**

**Tribe Lepidonotopodini Pettibone, 1983**

### Genus *Cladopolynoe* Hiley & Rouse in Hiley, Mongiardino Koch & Rouse, 2024

#### Cladopolynoe jiaolongae

Taxon classificationAnimaliaPhyllodocidaPolynoidae

(Han, Zhou, Chen & Wang, 2023)

6C7586A6-7E05-52BB-B26B-2287DCA63487

Figs 2, 3

Branchinotogluma jiaolongae Han, Zhou, Chen & Wang, 2023: fig. 5.
Cladopolynoe jiaolongae —Hiley et al. 2024: 21.

##### Material examined.

Indian Ocean • 1 ♂, 1 ♀; Onnuri; 11°24.96'S, 66°25.35'E; depth 2009 m; 1 Apr. 2023; collected by W-K Lee; hydrothermal vent; KRIBB314001–2 • 5 ♂, 1 ♀, 2 undetermined; Saero; 11°19.64'S, 66°26.88'E; depth 3273 m; 6 May 2024; collected by W-K Lee; hydrothermal vent; KRIBB314003–10.

**Figure 2.**
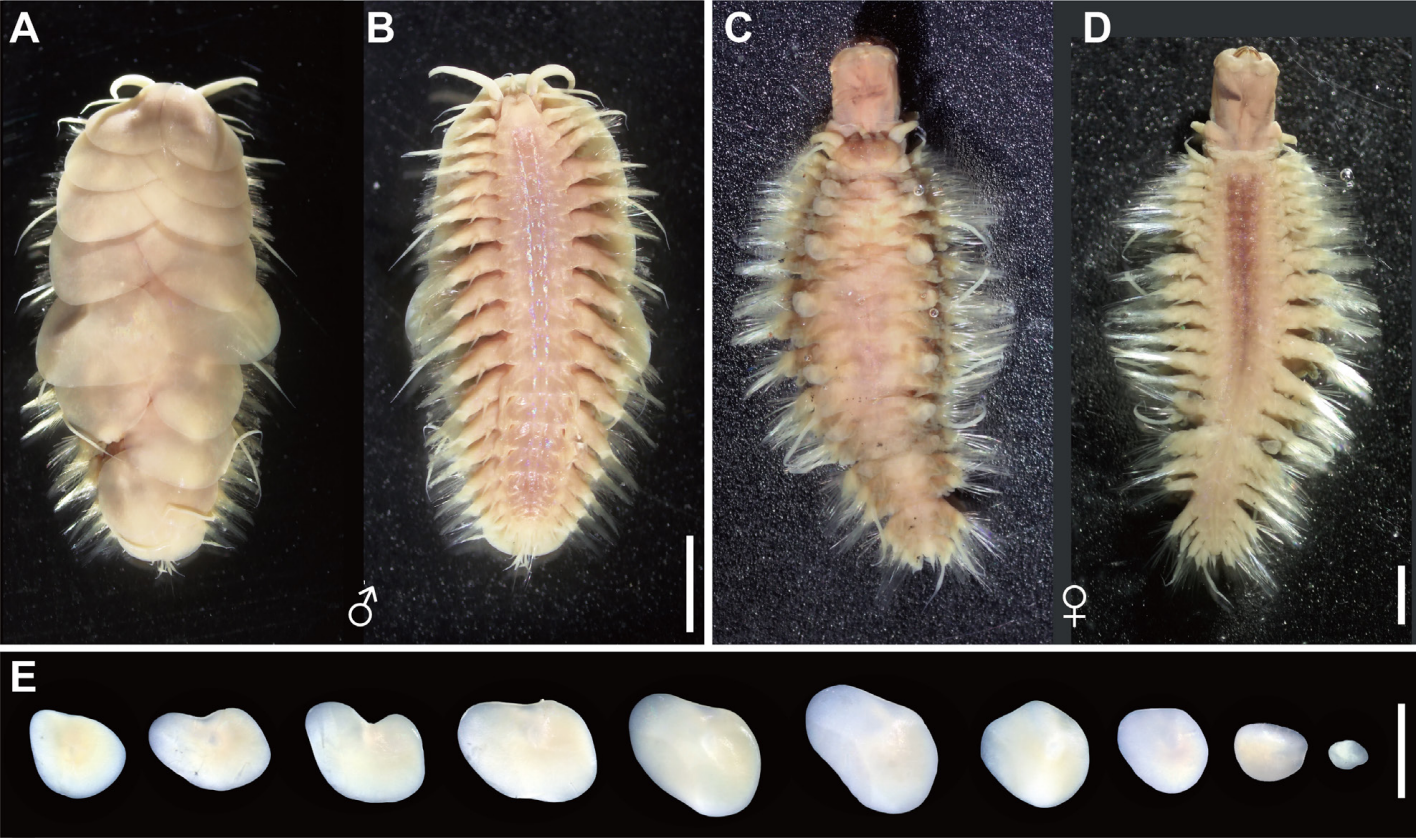
*Cladopolynoe jiaolongae* specimens collected from the nCIR. **A, B**. Dorsal and ventral views of a male; **C, D**. Dorsal and ventral views of female specimens; **E**. 1^st^–10^th^ right elytra of a male. Scale bars: 5 mm (**A, B, E**); 1 mm (**C, D**).

**Figure 3.**
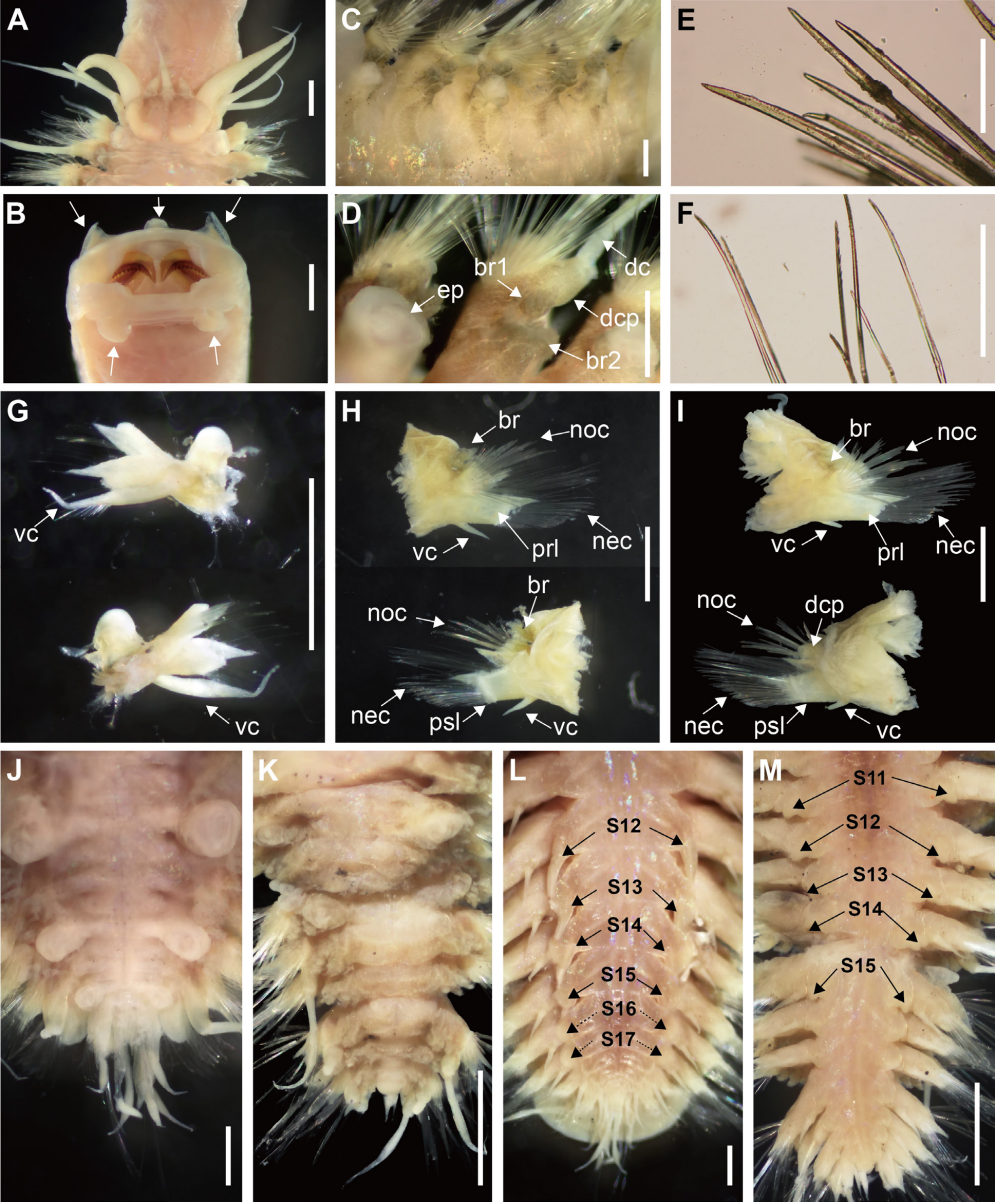
Micrographs of *Cladopolynoe jiaolongae* specimens from the nCIR. **A**. Head featuring prostomium, palps, tentacular cirri, and first two parapodia on segments 2 and 3; **B**. Everted pharynx with three dorsal and two ventral papillae pointed with arrows; **C**. Dorsal view of right segments 9–13; **D**. Branchiae, elytrophore and cirrophore on right segments 13–15; **E**. Notochaetae from segment 4; **F**. Neurochaetae from segment 3; **G–I**. Anterior and posterior views of right parapodia on segment 2, left parapodia on segments 9 and 10, respectively; **J, K**. Dorsal view of posterior segments of a male and female, respectively; **L, M**. Ventral view of posterior segments of a male and female, respectively. Solid arrows indicate ventral papillae and dotted arrows indicate ventral lamellae on each segment. Abbreviations: br, branchiae; dc, dorsal cirrus; dcp, cirrophore of dorsal cirrus; ep, elytrophore; nec, neurochaetae; noc, notochaetae; prl, prechaetal lobe of neuropodium; psl, postchaetal lobe of neuropodium; vc, ventral cirrus. Scale bars: 0.5 mm (**A–D, G–I**); 0.2 mm (**E, F**); 1 mm (**J–M**).

##### Diagnosis (emended).

*Cladopolynoe* found in CR, CIR, and SWIR. Ten pairs of elytra. Elytra oval to subreniform. Prostomium bilobed; paired tapering palps; lateral antennae absent; median antenna with cylindrical ceratophore in anterior notch; frontal filaments present. Pharynx with three dorsal and two ventral papillae. Two pairs of dorsal and ventral jaws, each with numerous teeth on inner border. Notopodia with arborescent branchiae in two groups, starting on segment 3 and continuing to segment 16, 18, or posterior end. Notochaetae with two rows of spines. Males with four pairs of long ventral papillae on segments 12–15 and two pairs of rounded ventral lamellae on segments 16–17. Females with five pairs of small, round ventral papillae on segments 11–15, ventral lamellae absent.

##### Description.

Body, fusiform and flattened with 21 segments, 5.8–27.0 mm in length and 3.0–14.0 mm in width (Fig. 2A–D). Elytra 10 pairs, present on dorsal side of segments 2, 4, 5, 7, 9, 11, 13, 15, 17, and 19, fully covering dorsum; shape oval or subreniform, smooth with branching veins (Fig. 2E). Dorsal cirri on non-elytragerous segments; long, extending beyond the neurochaetae; dorsal cirrophore cylindrical, slightly swollen (Figs 2C, 3A).

Prostomium oval, deeply bilobed (Fig. 3A). Anterior lobes subtriangular, with tapering frontal filaments; lateral antennae absent; median antenna inserted in anterior notch, tapering distally (Fig. 3A). Eyes absent. One pair of palps, stout and tapering distally (Fig. 3A). First segment fused to prostomium; two pairs of tentacular cirri, slender and tapering distally (Fig. 3A). Second segment, first biramous parapodia with first pair of elytra and bracts; ventral cirri longer than those in following segments (Fig. 2B, D). Mouth located ventrally, between first and second segments (Fig. 2B, D); pharynx with three dorsal and two ventral papillae (Fig. 3B); two pairs of jaws, each with numerous teeth on the inner border (Fig. 3B).

Parapodia biramous, from second to last segments. Branchiae present from segment 3 to 16, 18 or 21; arborescent form, in two clusters, one on lateral side of dorsal tubercle or elytrophore and other on base of dorsal cirri or the notopodia (Fig. 3C, D, J, K). Notopodia smaller than neuropodia; notochaetae thicker than neurochaetae, in radiating bundles (Fig. 3E, G–I). Neuropodia with triangular prechaetal lobe, rounded postchaetal lobe; neurochaetae numerous, in fan-shaped bundles (Fig. 3F, G–I). Pygidium rectangular, with long, slender anal cirri (Fig. 3L, M).

Sexual dimorphism present. In males, posterior segments modified; elytrophore and elytra on segment 19 small; branchiae present on segments 3 to 16 or 18; four pairs of ventral papillae, tapering with slender tips, positioned on segments 12–15; two pairs of ventral lamellae, round, positioned on segments 16 and 17 (Fig. 3J, L). In females, branchiae present on segments 3–21; five pairs of ventral papillae, tiny, round, present on segments 11–15; ventral lamellae absent (Fig. 3M).

##### DNA barcoding.

Ten new *COI* barcodes (586 bp; Table 1) were generated from the *C. jiaolongae* specimens collected at the nCIR and compared to sequences from the SWIR (*N* = 2; GenBank accession nos OQ784747 and OQ784749) and CR (*N* = 1; GenBank accession no. OQ784755). Intra-population variation was 0.00–0.34% for the nCIR population and 0.51% for the SWIR population (Suppl. material 1: table SS1). The inter-population divergence across all three regions was remarkably low at 0.20–0.31%. These values are substantially lower than the interspecific divergences within *Cladopolynoe* of 16.32–20.62%, indicating that *COI* barcodes can effectively discriminate species within this genus according to the barcoding threshold (Hebert et al. 2003) (Suppl. material 1: table S2).

##### Remarks.

*Cladopolynoe jiaolongae* has previously been recorded from the CR in the northern Indian Ocean and the SWIR in the south, two regions separated by approximately 6500 km along the ridge system (Han et al. 2023). In this study, we provide the first record of *C. jiaolongae* from the nCIR, effectively filling the distributional gap between the SWIR and CR populations.

Morphologically, the nCIR specimens generally correspond to the original description (Han et al. 2023). However, variation in the position of the last segment bearing branchiae was observed in the nCIR population. An emendation to the species diagnosis based on Han et al. (2023) is made to include specimens observed for this study with a smaller minimum number of branchiae (14 pairs, terminating on segment 16, instead of segment 21). Additionally, photographic data of elytral pairs from anterior to posterior are provided for the first time, fully characterizing elytral morphology, including their oval to subreniform shape and smooth surface.

Regarding molecular divergence, *C. jiaolongae* populations exhibited no significant genetic barriers, with *COI* barcode variation remaining within the intraspecific range (0.00–0.51%), far below the interspecific divergence of genus *Cladopolynoe* (16.32–20.62%).

##### Distribution.

Indian Ocean: Tiancheng vent field on the northern SWIR; Saero and Onnuri vent fields on the nCIR; Wocan vent field on the CR.

### Genus *Photinopolynoe* Hiley & Rouse in Hiley, Mongiardino Koch & Rouse, 2024

#### Photinopolynoe kaireiensis

Taxon classificationAnimaliaPhyllodocidaPolynoidae

(Han, Zhou, Chen & Wang, 2023)

56AF02E6-44E0-545D-AB06-24E38EB92700

Figs 4, 5

Branchinotogluma kaireiensis Han, Zhou, Chen & Wang, 2023: fig. 4.
Photinopolynoe kaireiensis —Hiley et al. 2024: 23.

##### Material examined.

Indian Ocean • 1 ♂, 1 ♀; Maru; 15°31.87'S, 67°17.90'E; depth 2256 m; 1 May 2024; collected by W-K Lee; hydrothermal vent; KRIBB315001–2 • 1 ♀; Cheoeum; 12°37.09'S, 66°7.58'E; depth 3038 m; 3 May 2024; collected by W-K Lee; hydrothermal vent; KRIBB315003 • 2 ♂, 2 ♀, 2 undetermined; Onnuri; 11°24.91'S, 66°25.43'E; depth 2013 m; 5 May 2024; collected by W-K Lee; hydrothermal vent; KRIBB315004–9 • 1 ♂, 2 ♀; Saero; 11°19.65'S, 66°26.88'E; depth 3267 m; 6 May 2024; collected by W-K Lee; hydrothermal vent; KRIBB315010–12 • 1 ♂, 4 ♀, 1 undetermined; Onbada; 9°48.96'S, 66°40.60'E; depth 2532 m; 7 May 2024; collected by W-K Lee; hydrothermal vent; KRIBB315013–18.

**Figure 4.**
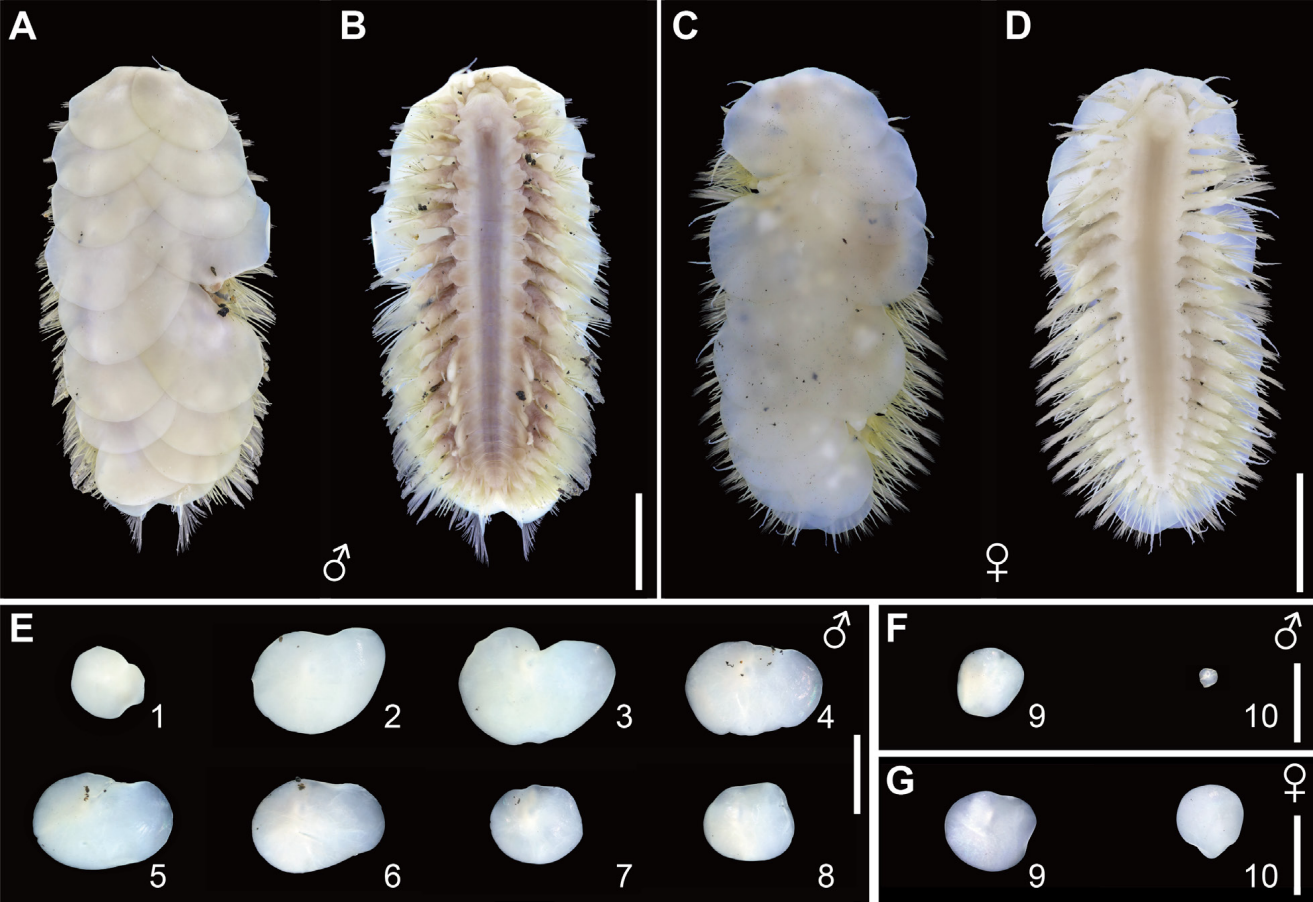
*Photinopolynoe kaireiensis* specimens collected from the nCIR. **A, B**. Dorsal and ventral views of a male; **C, D**. Dorsal and ventral views of female specimen; **E**. 1^st^–8^th^ left elytra; **F**. 9^th^–10^th^ left elytra of a male; **G**. 9^th^–10^th^ left elytra of a female. Scale bars: 5 mm.

**Figure 5.**
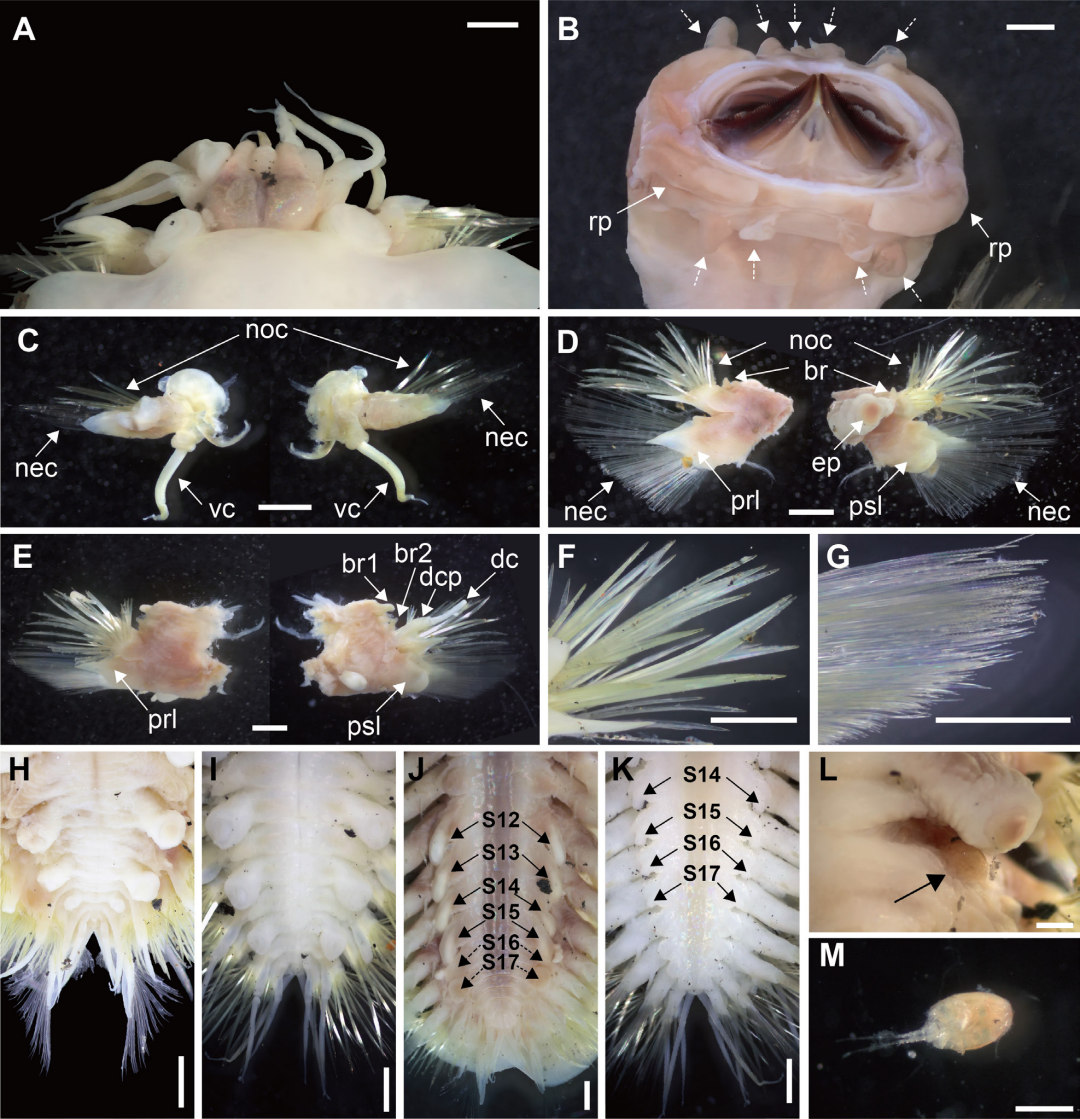
Micrographs of *Photinopolynoe kaireiensis* specimens from the nCIR. **A**. Head featuring prostomium, palps, tentacular cirri, and first parapodia on segment 2; **B**. Everted pharynx with five dorsal and four ventral papillae pointed with dotted arrows; **C–E**. Anterior and posterior views of right parapodia on segments 2, 9, and 12, respectively; **F, G**. Notochaetae and neurochaetae of segment 10, respectively; **H, I**. Dorsal view of posterior segments of a male and female, respectively; solid arrows indicate ventral papillae and dotted arrows indicate ventral lamellae on each segment; **J, K**. Ventral view of posterior segments of a male and female, respectively; **L**. A copepod between the elytrophore and body of *P. kaireiensis*; **M**. A copepod separated from the polynoid. Abbreviations: br, branchiae; dc, dorsal cirrus; dcp, cirrophore of dorsal cirrus; ep, elytrophore; nec, neurochaetae; noc, notochaetae; prl, prechaetal lobe of neuropodium; psl, postchaetal lobe of neuropodium; rp, rounded process; vc, ventral cirrus. Scale bars: 1 mm (**A–K**); 0.5 mm (**L, M**).

##### Diagnosis (emended).

*Photinopolynoe* found in CIR and SWIR. Ten pairs of elytra. Elytra oval to subreniform; smooth. Prostomium bilobed; paired tapering palps; lateral antennae absent; median antenna with cylindrical ceratophore in anterior notch; frontal filaments present. Pharynx with a pair of rounded processes at the lateral sides and five dorsal and four ventral papillae. Two pairs of dorsal and ventral jaws each with ca 30 teeth on the inner border. Notopodia with arborescent branchiae; present in 2 groups; starting on segment 3 and continuing to segment 16, 17, 18, 19, 20, or 21. Males with four pairs of long ventral papillae on segments 12–15 and two pairs of ventral lamellae on segments 16–17. Females with seven pairs of ventral papillae on segments 11–17. Females without ventral lamellae.

##### Description.

Body, fusiform and flattened with 21 segments, 10.3–41.0 mm in length and 6.0–18.0 mm in width (Fig. 4A–D). Elytra 10 pairs, present on dorsal side of segments, 2, 4, 5, 7, 9, 11, 13, 15, 17, and 19, fully covering dorsum; shape oval or subreniform, smooth with branching veins (Fig. 4E–G). Dorsal cirri on non-elytragerous segments; long, extending toward the tip of neurochaetae; dorsal cirrophore cylindrical (Figs 4D, 5E).

Prostomium oval, deeply bilobed (Fig. 5A). Anterior lobes subtriangular, with tapering frontal filaments; lateral antennae absent; median antenna inserted in anterior notch, tapering distally (Fig. 5A). Eyes absent. One pair of palps, stout and tapering, with short tip distally (Fig. 5A). First segment fused to prostomium; two pairs of tentacular cirri, slender and tapering distally with thin tips (Fig. 5A). Second segment, first biramous parapodia with first pair of elytra; ventral cirri longer than those in following segments (Fig. 5C). Mouth located ventrally, between first and second segments (Fig. 4B, D); pharynx with a pair of rounded processes at lateral bases, with five dorsal and four ventral papillae (Figs 4B, 4D, 5B); two pairs of jaws each with ca 30 teeth on the inner border (Fig. 5B).

Parapodia biramous, from second to the last segments. Branchiae present from segment 3 to 16, 17, 18, 19, 20, or 21; arborescent form, in two clusters, one on the lateral of dorsal tubercle or elytrophore and the other on base of dorsal cirri or the notopodia (Fig. 5D, E). Notopodia smaller than neuropodia; notochaetae thicker than neurochaetae, in radiating bundles. Neuropodia with triangular prechaetal lobe, rounded postchaetal lobe; neurochaetae thin and numerous, in fan-shaped bundles (Fig. 5C–G). Pygidium round with long and slender anal cirri (Fig. 5J, K).

Sexual dimorphism present. In males, parapodia of segment 18–21 reduced; elytrophore and elytra on segment 19 small; four pairs of ventral papillae positioned on segments 12–15, well developed with slender tips; two pairs of ventral lamellae, positioned on segments 16 and 17, round (Fig. 5H, J). In females, 7 pairs of ventral papillae positioned on segments 11–17, short and blunt, ventral lamellae absent (Fig. 5I, K). Three copepods were found under the elytra, between parapodia in one of the specimens (Fig. 5L, M).

##### DNA barcoding.

Eighteen new *COI* barcodes (579 bp; Table 1) were generated from the *P. kaireiensis* specimens collected at the nCIR and compared to sequences from the sCIR (*N* = 3; GenBank accession nos OQ784744–OQ784746) and CR (*N* = 1; GenBank accession no. OQ784758). Intra-population variation was 0.00–1.21% for the nCIR population and 0.17–1.55% for the sCIR population (Suppl. material 1: table S3). The inter-population divergence across all three regions was low at 0.37–1.09%. These values are substantially lower than the interspecific divergences within *Photinopolynoe* of 7.77–21.97%, indicating that *COI* barcodes can effectively discriminate species within this genus according to the barcoding threshold (Hebert et al. 2003) (Suppl. material 1: table S4).

**Remarks**. *Photinopolynoe kaireiensis* has previously been recorded from the sCIR and the CR in the Indian Ocean (Han et al. 2023). In this study, we provide the first record of this species from the nCIR, effectively filling the distributional gap between the sCIR and CR populations, which are separated by approximately 4000 km along the ridge system.

Morphologically, we observed a notable discrepancy in the number of pharyngeal papillae compared to the original description by Han et al. (2023). While they reported three dorsal and two ventral papillae from the sCIR population based on the specimen with dissected pharynx (Han et al. 2023: fig. 4G), our specimens from the nCIR consistently exhibited five dorsal and four ventral papillae, clearly observed from naturally everted pharynx (Fig. 5B). Because pharyngeal papillae are observed with greater accuracy and clarity when the pharynx is everted, the lower counts in the original description likely stemmed from the technical difficulties of observing an inverted, dissected structure. Given that the number of pharyngeal papillae is a stable diagnostic character in Polynoidae with rare intraspecific variation (Hiley et al. 2024), we consider these differences to be an artifact of observational methods rather than interpopulation variation. Therefore, an emendation of the species diagnosis by Han et al. (2023) is provided here to reflect this more accurate morphological characterisation. Additionally, photographic data of elytral pairs from anterior to posterior are provided for the first time, fully characterising elytral morphology, including their oval to subreniform shape and smooth surface.

Regarding molecular divergence, *P. kaireiensis* populations exhibited no significant genetic barriers, with *COI* sequence variation remaining within the intraspecific range (0.00–1.55%), far below the interspecific divergence of genus *Photinopolynoe* (7.77–21.97%).

Additionally, one specimen from the nCIR population was found with copepods between the parapodia beneath the elytra (Fig. 5L, M). While symbiotic relationships between polynoids and copepods have previously been documented (Leigh-Sharpe 1926; Stock 1996; Boxshall et al. 2019), the ecological relationship of vent-endemic *P. kaireiensis* and observed copepods remains unclear.

**Distribution**. Indian Ocean: Kairei vent field on the southern CIR; Onbada, Saero, Onnuri, Cheoeum, and Maru vent fields on the nCIR; Wocan vent field on the CR.

### Genus *Themis* Hiley & Rouse in Hiley, Mongiardino Koch & Rouse, 2024

#### Themis longqiensis

Taxon classificationAnimaliaAsparagalesAsparagaceae

(Han, Zhou, Chen & Wang, 2023)

44F31733-64E3-51CC-8057-A55D27D4A8AF

Figs 6, 7

Levensteiniella longqiensis Han, Zhou, Chen & Wang, 2023: 6, fig. 3.
Themis longqiensis —Hiley et al. 2024: 25.

##### Material examined.

Indian Ocean • 1 ♀; Onnare; 9°47.41'S, 66°41.89'E; depth 2991 m; 3 Apr. 2023; collected by W-K Lee; hydrothermal vent; KRIBB316001 • 1 ♂, 1 ♀; Onbada; 9°48.90'S, 66°40.62'E; depth 2563 m; 4 Apr. 2023; collected by W-K Lee; hydrothermal vent; KRIBB316002–3.

**Figure 6.**
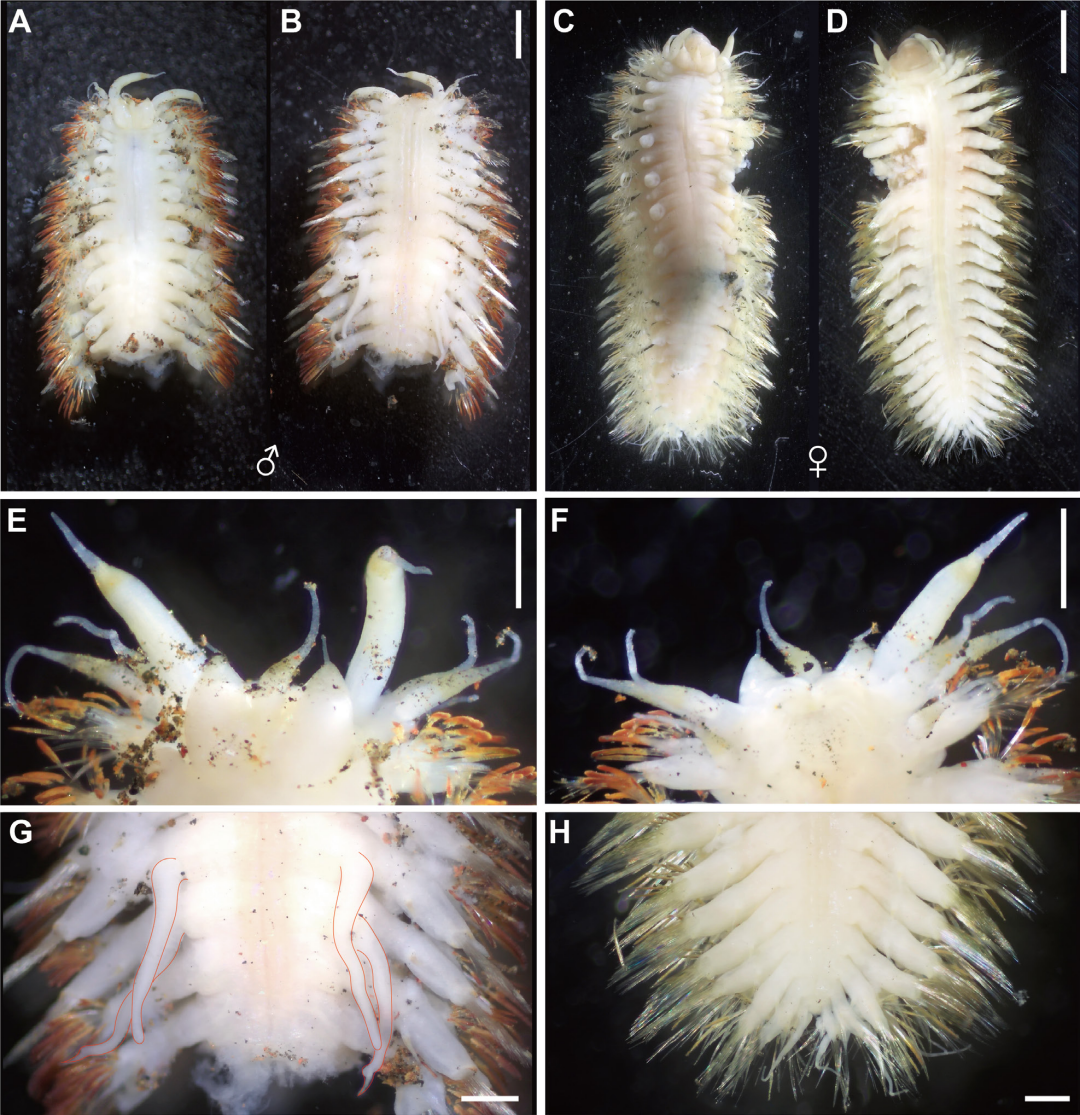
*Themis longqiensis* specimens collected from the nCIR. **A, B**. Dorsal and ventral views of male specimen; **C, D**. Dorsal and ventral views of female specimen; **E**. Head in dorsal view, featuring prostomium, palps, tentacular cirri, and first parapodia on segment 2; **F**. Head in ventral view, featuring ventral cirri on the first parapodia; **G**. Ventral view of segments 11–14 of a male with ventral papillae on segment 11–12 (outlined with red); **H**. Ventral view of posterior segments of a female. Scale bars: 2 mm (**C, D**); 1 mm (**A, B**); 0.5 mm (**E–H**).

**Figure 7.**
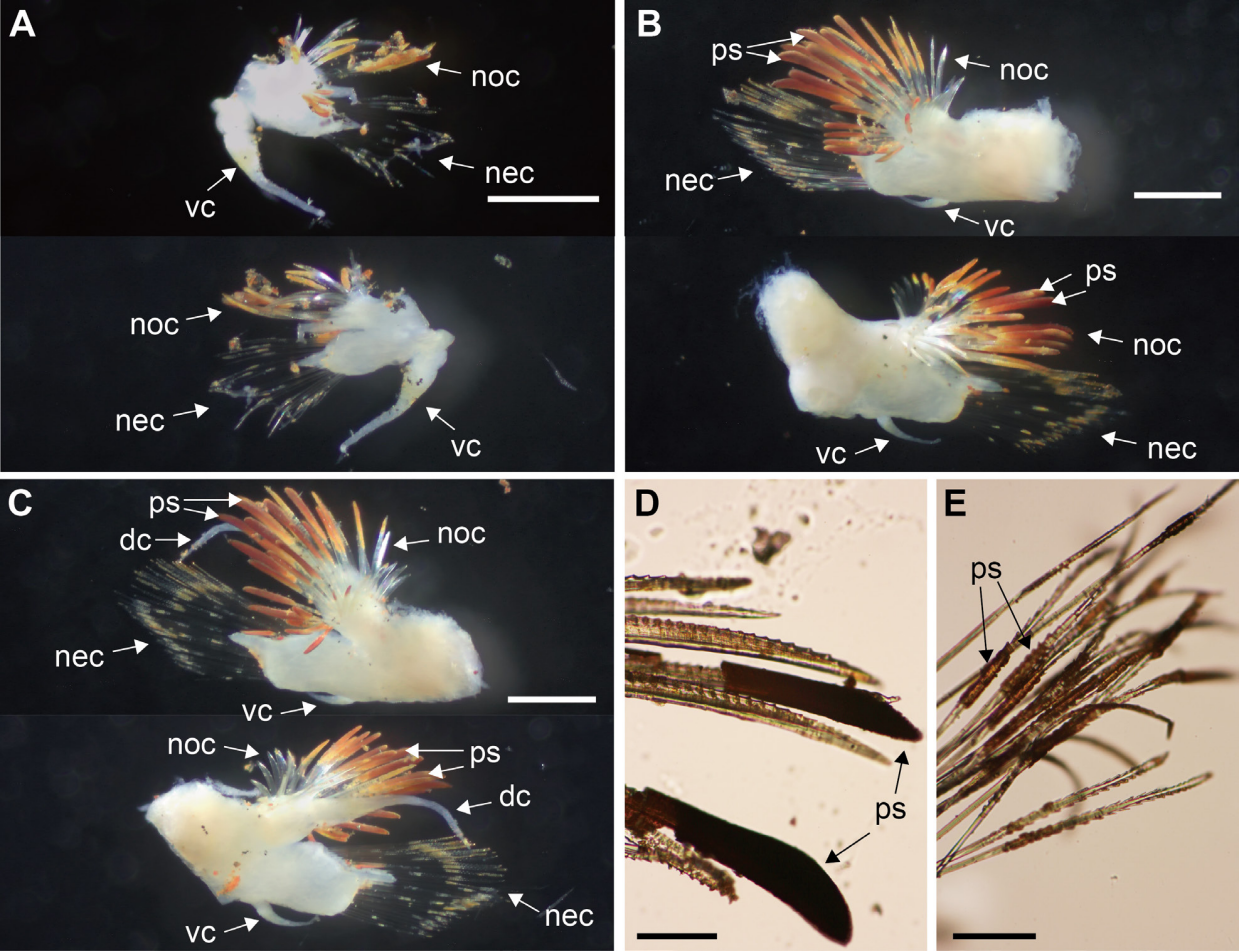
Micrographs of *T. longqiensis* specimens from the nCIR. **A–C**. Anterior and posterior views of left parapodia on segment 2, right parapodia on segments 9 and 10, respectively; **D, E**. Notochaetae and neurochaetae of segment 9, respectively. Abbreviations: **dc**, dorsal cirrus; **nec**, neurochaetae; **noc**, notochaetae; **ps**, polymetallic sulfide; **vc**, ventral cirrus. Scale bars: 0.5 mm (**A–C**); 0.1 mm (**D, E**).

##### Diagnosis (emended).

*Themis* found in SWIR and CIR, with 25 segments and 11 pairs of elytrophores. Prostomium bilobed; paired tapering palps; lateral antennae absent; median antenna with cylindrical ceratophore in anterior notch, frontal filaments present. Two pairs of dorsal and ventral jaws, smooth, without teeth. Notochaetae stout, with serrated distal parts and pointed tips, covered with polymetallic sulfide. Neurochaetae long and slender, with two rows of widely spaced acicular spines and hooked tip. Males with two pairs of long ventral papillae on segments 11–12, ventral papillae on segment 12 spiraled at the distal tip. Females without ventral papillae.

##### Description.

Body, fusiform and flattened with 25 segments, 13.9 mm in length and 4.1 mm in width (Fig. 6C, D). Elytra missing, elytrophores on segments, 2, 4, 5, 7, 9, 11, 13, 15, 17, 19, and 21 (Fig. 6C). Dorsal cirri on non-elytragerous segments; long, extending beyond notochaetae (Figs 6C, 7C).

Prostomium oval, deeply bilobed (Fig. 6E). Anterior lobes subtriangular, with long frontal filaments; lateral antennae absent; median antenna, conical and tapering distally, inserted in anterior notch (Fig. 6E). Eyes absent. One pair of palps, stout, tapering, with thin tip distally (Fig. 6E, F). First segment fused to prostomium; two pairs of tentacular cirri slender and tapering distally with thin tips (Fig. 6E, F). Second segment, with first biramous parapodia and first pair of elytra; ventral cirri longer than those in following segments (Figs 6F, 7A). Mouth located ventrally, between first and second segments (Fig. 6F); two pairs of jaws, smooth, without teeth.

Parapodia biramous, from second to the posteriormost segments. Notopodia smaller than neuropodia; notochaetae in radiating bundles, stout, thicker than neurochaetae, serrated in distal part, covered with thick polymetallic sulfide at the tip (Fig. 7A–D). Neuropodia with acicular prechaetal lobe, rounded postchaetal lobe; neurochaetae in fan-shaped bundles, long and slender, numerous, with two rows of widely spaced acicular spines and hooked tip, some covered with a thin layer of polymetallic sulfide (Fig. 7A–C, E). Pygidium round with long and slender anal cirri (Fig. 6H). Branchiae absent. Ventral lamellae absent.

Sexual dimorphism present. In males, two pairs of elongate, tapering ventral papillae positioned on segments 11 and 12, ventral papillae on segment 12 spiraled at distal tip (Fig. 6G). In females, ventral papillae absent.

##### DNA barcoding.

Three new *COI* barcodes (606 bp; Table 1) were generated from the *T. longqiensis* specimens collected at the nCIR and compared to sequences from the SWIR (*N* = 2; GenBank accession nos OQ784752 and OQ784753). Intra-population variation was 0.00–0.33% for the nCIR population and 0.99% for the SWIR population (Suppl. material 1: table S5). The inter-population divergence between the SWIR and nCIR populations was low at 0.61%. These values are substantially lower than the interspecific divergences within *Themis* of 12.59–24.47%, indicating that *COI* barcodes can effectively discriminate species within this genus according to the barcoding threshold (Hebert et al. 2003) (Suppl. material 1: table S6).

**Remarks**. *Themis longqiensis* has previously been recorded only from the SWIR (Han et al. 2023). In this study, we provide the first record of *T. longqiensis* from Onnare and Onbada vent fields, expanding its distribution to the nCIR. Compared to other hydrothermal vent organisms, the adults of this species are relatively small (approximately 1 cm) and fragile, making them prone to being overlooked during surveys.

Morphologically, the specimens obtained from the nCIR are more complete than those in the original description (Han et al. 2023). Therefore, an emendation of species diagnosis by Han et al. (2023) is provided here to reflect the improved description of *T. longqiensis*, including details on the number of segments, the number of elytrophores, and the morphology of the pygidium and anal cirri (Fig. 6). Furthermore, sexual dimorphism was fully documented and photographed for the first time (Fig. 6G, H). Specifically, the presence of long ventral papillae in males, previously only partially described due to the truncated nature of the original specimens, was confirmed. In females, the absence of ventral papillae was also verified, resolving the uncertainty in the original description (Han et al. 2023). Despite these additions, the morphology of the elytra remains unknown, due to their small size and tendency to detach during sampling.

Regarding molecular divergence, *T. longqiensis* populations exhibited no significant genetic barriers, with *COI* sequence variation remaining within the intraspecific range (0.00–1.32%). However, as the number of individuals analyzed remains small, further studies involving additional specimens from the nCIR, SWIR, and intermediate regions (such as the sCIR) are necessary to fully characterize the genetic diversity and population structure of this species.

**Distribution**. Indian Ocean: Longqi vent field on the southern SWIR; Onnare and Onbada vent fields on the nCIR.

## Discussion

This study documents new records of three vent-endemic Lepidonotopodini species from the nCIR, filling a distributional gap between the vent fields in the southern and northern Indian Ocean. By examining a larger number of specimens than previous studies, we document a broader range of intraspecific morphological variability for these taxa. This expanded morphological dataset serves as a valuable reference for future taxonomic identification, reducing the risk of misidentification in understudied deep-sea populations. Additionally, integrating morphological data with DNA barcoding improves species identification and updates their biogeographic ranges for these taxa, enhancing our understanding of species diversity and connectivity within Indian Ocean hydrothermal vent ecosystems.

Hydrothermal vent fauna typically exhibits high endemism and fragmented distributions due to the patchy nature of vent habitats. However, some taxa exhibit remarkable biogeographic connectivity across expansive ridge systems. The presence of these Lepidonotopodini species from multiple vent fields in the nCIR enhances our understanding of regional vent diversity and presents the faunal similarity between geographically distant vent fields, supporting the hypothesis that the nCIR acts as a “stepping stone” facilitating dispersal across the Indian Ocean ridge systems.

The genetic evidence aligns with these biogeographic patterns. While the average intraspecific *COI* variation for polychaetes is approximately 3.92% (Kvist 2016), the examined Lepidonotopodini species exhibited significantly lower variation (0.00–1.55%), despite population separation exceeding approximately 6500 km. This relative genetic homogeneity implies recent range expansions or sustained gene flow across the Indian Ocean. Potential mechanisms facilitating this connectivity include planktonic larval dispersal and the utilization of intermediate vent fields along the ridge. The topographic continuity of the ridge system, combined with deep-sea currents, may further enhance the larval dispersal between geographically isolated populations.

In conclusion, our results highlight the potential for extensive connectivity of hydrothermal vent fauna in the Indian Ocean, emphasizing the need for further studies and sampling to fully elucidate the complex biogeographic patterns and evolutionary history of the Indian Ocean vent fauna.

## Supplementary Material

XML Treatment for Cladopolynoe jiaolongae

XML Treatment for Photinopolynoe kaireiensis

XML Treatment for Themis longqiensis

## Additional information

### Artificial Intelligence (AI) use

The authors accept full responsibility for the content of the manuscript, including the disclosure of any use of AI.

No AI tools were used in the preparation of this manuscript.

### Author ORCIDs

Won-Kyung Lee https://orcid.org/0000-0001-7283-298X

Se-Joo Kim https://orcid.org/0000-0003-1653-072X

### Data availability

All of the data that support the findings of this study are available in the main text or Supplementary Information.

## Supplementary materials

Supplementary material 1 Sequence divergence between the *COI* barcodes of each species and genus Data type xlsx Explanation note Intraspecific sequence divergences among *COI* barcodes are presented for *Cladopolynoe jiaolongae*, *Photinopolynoe kaireiensis*, and *Themis longqiensis*, along with interspecific divergences within each genus. https://binary.pensoft.net/file/1737978This dataset is made available under the Open Database License (http://opendatacommons.org/licenses/odbl/1.0/). The Open Database License (ODbL) is a license agreement intended to allow users to freely share, modify, and use this Dataset while maintaining this same freedom for others, provided that the original source and author(s) are credited. Won-Kyung Lee, Se-Joo Kim
